# Dickkopf-related protein 3 promotes pathogenic stromal remodeling in benign prostatic hyperplasia and prostate cancer

**DOI:** 10.1002/pros.22691

**Published:** 2013-06-14

**Authors:** Christoph Zenzmaier, Natalie Sampson, Eugen Plas, Peter Berger

**Affiliations:** 1 Institute for Biomedical Aging Research, University of InnsbruckInnsbruck, Austria; 2 Department of Internal Medicine, Innsbruck Medical UniversityInnsbruck, Austria; 3 Department of Urology, Innsbruck Medical UniversityInnsbruck, Austria; 4 Department of Urology, Hanusch HospitalVienna, Austria

**Keywords:** angiogenic factors, angiopoietin, myofibroblast differentiation, proliferation

## Abstract

**BACKGROUND** Compartment-specific epithelial and stromal expression of the secreted glycoprotein Dickkopf-related protein (Dkk)-3 is altered in age-related proliferative disorders of the human prostate. This study aimed to determine the effect of Dkk-3 on prostate stromal remodeling that is stromal proliferation, fibroblast-to-myofibroblast differentiation and expression of angiogenic factors in vitro.

**METHODS** Lentiviral-delivered overexpression and shRNA-mediated knockdown of *DKK3* were applied to primary human prostatic stromal cells (PrSCs). Cellular proliferation was analyzed by BrdU incorporation ELISA. Expression of Dkk-3, apoptosis-related genes, cyclin-dependent kinase inhibitors and angiogenic factors were analyzed by qPCR, Western blot analysis or ELISA. Fibroblast-to-myofibroblast differentiation was monitored by smooth muscle cell actin and insulin-like growth factor binding protein 3 mRNA and protein levels. The relevance of Wnt/β-catenin and PI3K/AKT signaling pathways was assessed by cytoplasmic/nuclear β-catenin levels and phosphorylation of AKT.

**RESULTS** Knockdown of *DKK3* significantly attenuated PrSC proliferation as well as fibroblast-to-myofibroblast differentiation and increased the expression of the vessel stabilizing factor angiopoietin-1. *DKK3* knockdown did not affect subcellular localization or levels of β-catenin but attenuated AKT phosphorylation in PrSCs. Consistently the PI3K/AKT inhibitor LY294002 mimicked the effects of *DKK3* knockdown.

**CONCLUSIONS** Dkk-3 promotes fibroblast proliferation and myofibroblast differentiation and regulates expression of angiopoietin-1 in prostatic stroma potentially via enhancing PI3K/AKT signaling. Thus, elevated Dkk-3 in the stroma of the diseased prostate presumably regulates stromal remodeling by enhancing proliferation and differentiation of stromal cells and contributing to the angiogenic switch observed in BPH and PCa. Therefore, Dkk-3 represents a potential therapeutic target for stromal remodeling in BPH and PCa. *Prostate 73: 1441–1452, 2013*. © 2013 The Authors. Prostate published by Willey-Blackwell. This is an open access article under the terms of the Creative Commons Attribution-Non-Commercial-NoDerivs Licence, which permits use and distribution in any medium, provided the original work is properly cited, the use is non-commercial and no modifications or adaptations are made.

## INTRODUCTION

Expression patterns of the secreted glycoprotein Dickkopf-related protein 3 (Dkk-3) are altered in benign prostatic hyperplasia (BPH) and prostate cancer (PCa). In the normal prostate, Dkk-3 is predominantly expressed in the epithelial compartment, whereas in the diseased prostate, Dkk-3 is reduced in the epithelial but elevated in the stromal compartment, especially endothelial cells 1. We recently demonstrated that in patients with PCa seminal plasma Dkk-3 levels are significantly elevated 2 despite the reduced expression of Dkk-3 in secretory epithelial cells 1,3 indicating that Dkk-3 derived from tumor neovasculature/stroma is able to diffuse through the diseased tissue into the prostatic fluid while in normal/BPH tissue an intact basement membrane prevents leakage from the stroma to the epithelial compartment and vice versa.

The functional significance of elevated Dkk-3 in the diseased prostatic stroma is unknown. Dkk-3 is the most divergent member of the human Dickkopf family of Wnt/β-catenin signaling antagonists 5–6. In contrast to other family members, Dkk-3 does not interact with LDL-receptor-related protein 6 or Kremen proteins and thus is not considered a Wnt/β-catenin signaling antagonist 7–8. Nevertheless, Dkk-3 has been reported to either antagonize 9–10 or enhance 11 Wnt/β-catenin signaling in a cell-specific manner.

Dkk-3 has been proposed to represent a novel tumor suppressor since gene expression is downregulated in various tumor cells 3–15 and hypermethylation of its promoter correlates with cancer occurrence 16–17. Several studies reported anti-proliferative or pro-apoptotic effects upon *DKK3* overexpression 3–19, However, these effects appeared to be caused by endoplasmatic reticulum stress (unfolded protein response) 18–19, which is commonly induced by overexpression of highly-glycosylated secreted proteins, such as Dkk-3, and thus might not reflect the biological role of endogenous Dkk-3. Indeed, addition of exogenous recombinant Dkk-3 uniformly failed to reduce proliferation or induce apoptosis of malignant and nonmalignant cells 1,19. Moreover, in the human pancreatic carcinoma cell line PANC-1 overexpression of *DKK3* did not alter cellular proliferation, while knockdown of *DKK3* resulted in significant reduction of cellular proliferation and concomitant induction of pancreatic epithelial cell differentiation markers, indicating that Dkk-3 is required to maintain a highly dedifferentiated and proliferative state in these cells 21.

BPH and PCa are both associated with changes in the stromal microenvironment (stromal remodeling) that actively promote disease development. In particular, the BPH and PCa-adjacent stroma are characterized by increased extracellular matrix deposition, capillary density, and differentiation of fibroblasts into myofibroblasts, the mitogenic secretome of which promotes proliferation, angiogenesis, and tumorigenesis 22–25. TGFβ1 is considered to be a key inducer of pathogenic stromal reorganization, and others and we have demonstrated that TGFβ1 induces prostatic fibroblast-to-myofibroblast differentiation 26–30.

Enhanced angiogenesis is also a key feature of the remodeled stroma. The angiogenic switch is a rate-limiting step in tumor progression 31 that is associated with a shift in the ratio of the vessel stabilizing angiopoietin-1 (*ANGPT1*) to the destabilizing factor angiopoietin-2 (*ANGPT2*) in favor of *ANGPT2*. Consequently, the angiogenic switch renders the tumor vasculature amenable to vessel sprouting 32. Besides the prostate elevated Dkk-3 expression has also been shown in vessels from other tumors for example in colorectal cancer, glioma, non-Hodgkin lymphoma, melanoma, and pancreatic adenocarcinoma whereas vessels from normal tissue express low/not detectable Dkk-3 levels 33,34. Dkk-3 has been shown to support tube formation in primary endothelial colony-forming cells and *DKK3* overexpression reduced *ANGPT1* expression in a murine B16F10 melanoma model 34. Moreover, Dkk-3 and *ANGPT2* were inversely regulated in human umbilical vein endothelial cells after knockdown of Axl 36, suggesting a role of Dkk-3 in tumor angiogenesis.

This study aimed to investigate the functional significance of elevated stromal Dkk-3 in BPH and PCa by lentiviral-delivered overexpression and shRNA-mediated knockdown of *DKK3* in primary prostatic stromal cells and analysis of the downstream effects on proliferation, TGFβ1-induced fibroblast-to-myofibroblast differentiation and expression of angiogenic factors.

## MATERIALS AND METHODS

### Cell Culture and Fibroblast-to-Myofibroblast Differentiation

Human primary prostatic stromal cell (PrSC) and prostatic basal epithelial cell (PrEC) cultures were established as described previously 1. PrSC were cultured in stromal cell growth medium (Quantum 333, PAA Laboratories), PrEC on collagen I-coated plates in prostate epithelial cell growth medium (PrEGM, Clonetics). All experiments were performed with primary cells from at least three independent donors. Fibroblast-to-myofibroblast differentiation was induced by 1 ng/ml TGFβ1 (R&D Systems) in RPMI 1640 (PAA Laboratories) containing 1% charcoal treated fetal calf serum (HyClone) and 1% penicillin/streptomycin (PAA Laboratories) as described 28. Control cells were treated with 1 ng/ml human basic fibroblast growth factor (bFGF; Sigma–Aldrich) as control to maintain the fibroblast phenotype.

PC3 and HT-29 cells were purchased from the American Type Culture Collection (ATCC). PC3 cells were cultured in RPMI 1640 (PAA Laboratories) containing 1% penicillin/streptomycin (PAA Laboratories) and 3% bovine calf serum (HyClone), HT-29 cells in MEM Eagle (PAN Biotech) containing 10% bovine calf serum and 1% penicillin/streptomycin, respectively.

### Knockdown and Overexpression of *DKK3* by Lentiviral Particles

Production of lentiviral particles was carried out according to the manufacturer's protocol (Addgene) as described previously 21 using the lentiviral pLKO.1-TRC short hairpin system (Addgene) for knockdown and full-length cDNA of *DKK3* subcloned into the pLenti6 vector (Invitrogen) for overexpression, respectively. The scramble shRNA vector (Addgene plasmid 1864) and the empty pLenti6 vector were used as controls. For viral transduction, cells were seeded in appropriate vessels and left to adhere overnight. Thereafter, medium was replenished and supplemented with virus-containing supernatant at MOI 4 (knockdown) and MOI 0.5 (overexpression), respectively.

For small interfering RNA (siRNA)-mediated *DKK3* knockdown PrSCs were seeded in 6-cm dishes and transfected with three different siRNA duplexes targeting *DKK3* (DKK3-siRNA#1: catalog no. HSS146900; DKK3-siRNA#2: catalog no. HSS146901; DKK3-siRNA#3: catalog no. HSS146899; Invitrogen) or scrambled control (catalog no. 12935-300; Invitrogen) using Lipofectamin 2000 (Invitrogen) according to manufacturer's instructions. Seventy-two hours after transfection, fibroblast-to-myofibroblast differentiation experiments were started.

### Cell Proliferation Assay

Two thousand cells were seeded in triplicate into 96-well plates (Nunc) in 100 µl culture medium and left to adhere overnight. Thereafter, fresh medium was supplemented with lentivirus particles to transduce cells or the phosphatidylinositol 3-kinase **(**PI3K) inhibitor LY294002 (Calbiochem) at the indicated concentration. Proliferation was determined by relative quantification of DNA synthesis using a bromodeoxyuridine (BrdU) cell proliferation ELISA (Roche Applied Science) according to the manufacturer's instructions at indicated times post-transduction.

### Quantitative Real-Time PCR

mRNA extraction, cDNA synthesis and quantitative PCR (qPCR) were performed as described elsewhere 28. Primer sequences are given in Table1. cDNA concentrations were normalized by the housekeeping gene hydroxymethylbilane synthase (*HMBS*).

**Table I:** Primer Sequences

Gene	Unigene ID	Primer sequences			
		Sense	Antisense
*ACTG2 (SMA)*	Hs.403989	5-agaagagctatgagctgcca	5-gctgtgatctccttctgcat
*ANGPT1*	Hs.369675	5-ctgatcttacacggtgctga	5-acaagcatcaaaccaccatc
*ANGPT2*	Hs.583870	5-aataagcagcatcagccaac	5-tcaagttggaaggaccacat
*CDKN1A* (p21^CIP1^)	Hs.370771	5-ggcggcagaccagcatgacagatt	5-gcagggggcggccagggtat
*CDKN1B* (p27^KIP1^)	Hs.238990	5-aataaggaagcgacctgcaa	5-cgagctgtttacgtttgacg
*DKK3*	Hs.292156	5-tcatcacctgggagctagag	5-caacttcatactcatcgggg
*HMBS*	Hs.82609	5-ccaggacatcttggatctgg	5-atggtagcctgcatggtctc
*IGFBP3*	Hs.450230	5-caagcgggagacgaatatg	5-ttatccacacaccagcagaa

### Dkk-3 and Angiopoietin Quantification in Cell Culture Supernatants

PrSCs were seeded at a density of 1 × 10^5^ per 6 cm dishes and left to adhere overnight. Subsequently, medium was replaced and cells were transduced with lentiviral particles. After 72 hr, medium was replaced with fresh medium containing bFGF, TGFβ1 and/or LY294002 as indicated, and conditioned for 24 hr (for determination of Dkk-3) and 72 hr (for determination of angiopoietin-1 and angiopoietin-2), respectively. Secreted Dkk-3 was quantified by immunoenzymometric assay (IEMA) as previously described 37–38. Secreted angiopoietin-1 and angiopoietin-2 levels were analyzed by the RayBio® Human angiopoietin-1 ELISA Kit and RayBio® Human angiopoietin-2 ELISA Kit (RayBiotech) according to manufacturer's instructions, respectively. In order to account for different cell proliferation angiopoietin levels were normalized using corresponding relative BrdU-incorporation ELISA values.

### Western Blot Analysis

Total cell extracts were prepared and analyzed by Western blot as described previously 1. Subcellular fractionation was performed using the Pierce NE-PER nuclear and cytoplasmic extraction reagents according to the manufacturer's instructions. Primary antibodies were obtained as follows: phospho-p53, p21^CIP1^, p27^KIP1^, phospho-AKT (Ser473) and phospho-Smad2 (Ser465/467) (Cell Signaling Technology); phospho-JNK (Thr183/Thr185) (Santa Cruz); LDH (Rockland); Dkk-3 and IGF binding protein (IGFBP)3 (R&D Systems); Bcl-2–associated X protein (BAX, Oncogene); β-catenin (Upstate Biotechnology); lamin B (Calbiochem); SMA and β-actin (Sigma–Aldrich); glyceraldehyde 3-phosphate dehydrogenase (GAPDH, Abcam).

### Immunofluorescence

Immunofluorescence for smooth muscle cell α-actin (SMA) was performed as described previously 27.

### Statistics

Results are expressed as mean values ± SEM. Statistical differences between treatments were calculated by paired Student's *t*-test and considered significant when *P* < 0.05 (*P < 0.05, **P < 0.01, ***P < 0.001).

## RESULTS

### Efficient Overexpression and Knockdown of Dkk-3 in PrSCs

**Figure 1:**
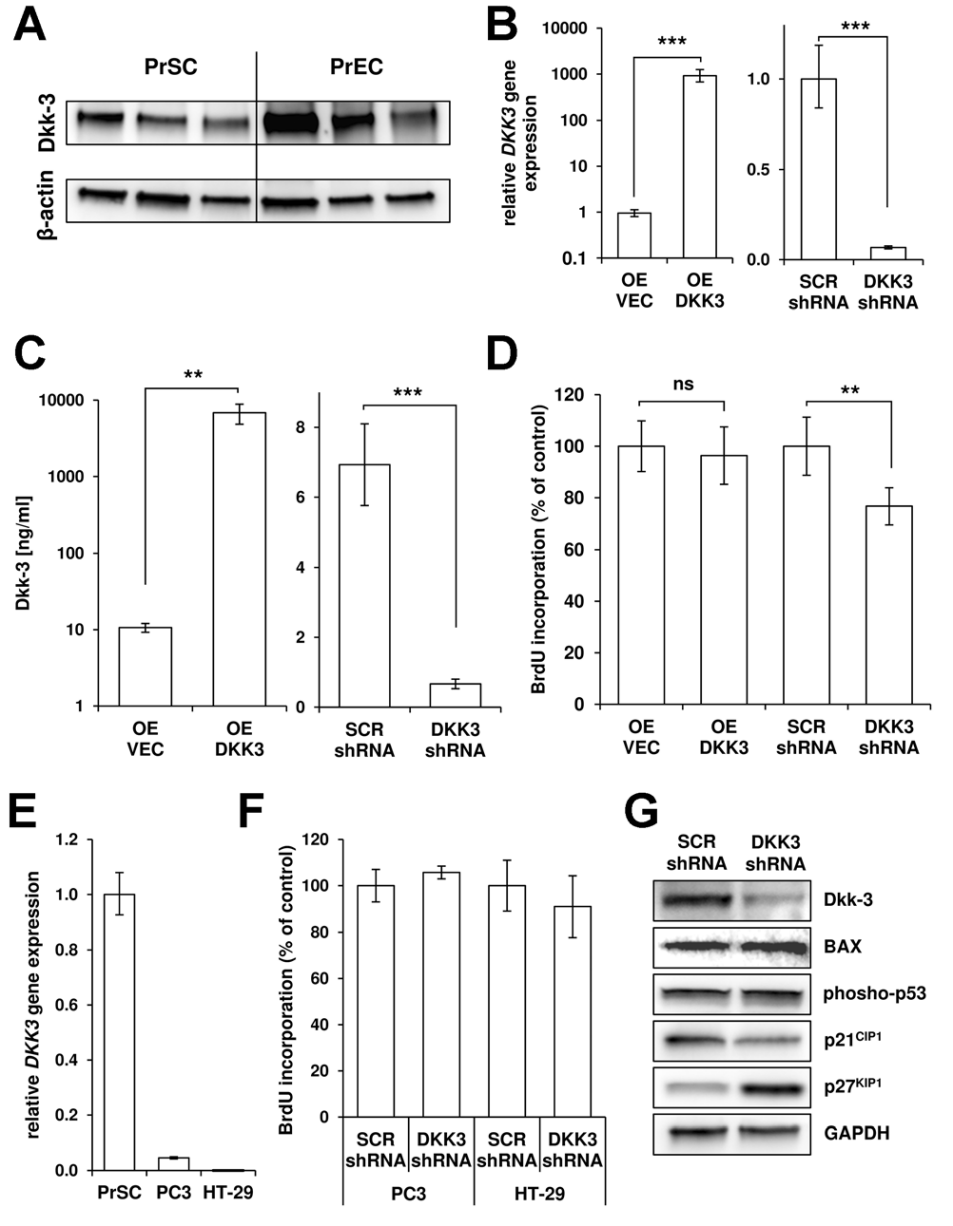
*DKK3* knockdown reduces PrSC proliferation and induces p27^KIP1^ levels. A: Western blot analysis of total cell lysates of primary prostatic stromal (PrSC) and epithelial (PrEC) cells isolated from three individual donors revealed significant Dkk-3 expression in both cell types. β-actin served as loading control. B: *DKK3* mRNA levels after lentiviral-delivered overexpression (OE DKK3) compared with empty vector control virus (OE VEC) and lentiviral-delivered *DKK3* specific shRNA (DKK3 shRNA) compared with scrambled control (SCR shRNA) as determined by qPCR 72h post-transduction of PrSCs (overexpression: n = 4; shRNA: n = 5). *DKK3* gene expression levels were normalized using the housekeeping gene *HMBS* and are shown relative to controls. C: Secreted Dkk-3 protein levels in PrSCs after overexpression and knockdown of *DKK3* (n = 4). D: DKK3-shRNA significantly reduced cellular proliferation of PrSCs determined by BrdU-incorporation ELISA at day 6 post-transduction (n = 5). E: *DKK3* mRNA levels of PC3 (n = 3) and HT-29 cells (n = 3) compared with PrSCs (n = 5). F: Cellular proliferation of PC3 and HT-29 cells as determined by BrdU-incorporation ELISA at day 6 post-transduction with DKK3-shRNA compared with SCR-shRNA, respectively (n = 3). G: Western blot analysis of apoptosis-related proteins (BAX, phospho-p53) and the cyclin-dependent kinase inhibitors p27^KIP1^ and p21^CIP1^ in DKK3-shRNA and SCR-shRNA PrSCs 72 hr post-transduction. GAPDH served as loading control.

Primary prostatic stromal cells (PrSCs) were used to investigate the functional significance of Dkk-3 in the stromal compartment in vitro. Consistent with the predominant expression of Dkk-3 in the epithelial compartment of the benign prostate 1, Dkk-3 was more abundant in cell lysates from primary prostatic epithelial cells (PrECs) than PrSCs at the protein level as determined by Western blot analysis (Fig. 1A), however PrSCs secreted Dkk-3 at significant levels (Fig. 1C).

To efficiently modify the expression of Dkk-3 in PrSCs in vitro, lentiviral-delivered *DKK3* overexpression and knockdown systems were established. Stable overexpression of *DKK3* resulted in approximately 10^3^-fold increase in *DKK3* mRNA (Fig. 1B; *P* = 0.0007) and 650-fold increase in secreted Dkk-3 protein levels (Fig. 1C; *P* = 0.006) compared with empty vector control, while knockdown by lentiviral-delivered shRNA targeting *DKK3* (DKK3-shRNA) significantly decreased Dkk-3 mRNA (Fig. 1B; 93% reduction; *P* = 0.0002) and secreted protein levels (Fig. 1C; 90% reduction; *P* = 0.0002) compared with scrambled control shRNA (SCR-shRNA).

### Dkk-3 Promotes Proliferation of PrSCs

We next investigated the influence of *DKK3* overexpression and knockdown on the proliferation of PrSCs in vitro. Consistent with previous observations using adenovirus-delivered transient *DKK3* overexpression 1, stable overexpression of *DKK3* did not influence proliferation of PrSCs (Fig. 1D). On the other hand, lentiviral-delivered DKK3-shRNA significantly reduced cellular proliferation of PrSCs by approximately 23% compared with the SCR-shRNA (Fig. 1D; *P* = 0.007). To exclude potential off-target effects of the lentiviral knockdown system the effect of DKK3-shRNA on proliferation was additionally investigated in PC3 prostate cancer and HT-29 colon carcinoma cell lines with low endogenous *DKK3* expression (Fig. 1E). DKK3-shRNA did not significantly affect cellular proliferation of both cell lines compared with SCR-shRNA control cells (Fig. 1F).

Knockdown of *DKK3* has been reported to induce apoptosis and increase levels of BAX, p53 and p21^CIP1^ in H460 lung cancer cells 39. Thus, levels of these markers and the cyclin-dependent kinase inhibitor p27^KIP1^ were analyzed in DKK3-shRNA PrSCs. Neither BAX nor phospho-p53 protein levels were significantly altered compared with SCR-shRNA, indicating that the lentiviral knockdown of *DKK3* did not induce apoptosis in PrSCs (Fig. 1G). Consistent with reduced proliferation of DKK3-shRNA PrSCs, *CDKN1A* (p21^CIP1^) and *CDKN1B* (p27^KIP1^) mRNA levels were significantly elevated compared to SCR-shRNA (Supplemental Fig. 1). However, at the protein level only p27^KIP1^ was elevated in DKK3-shRNA PrSCs, while p21^CIP1^ protein levels were found decreased (Fig. 1G).

### Dkk-3 Supports Fibroblast-to-Myofibroblast Differentiation

**Figure 2:**
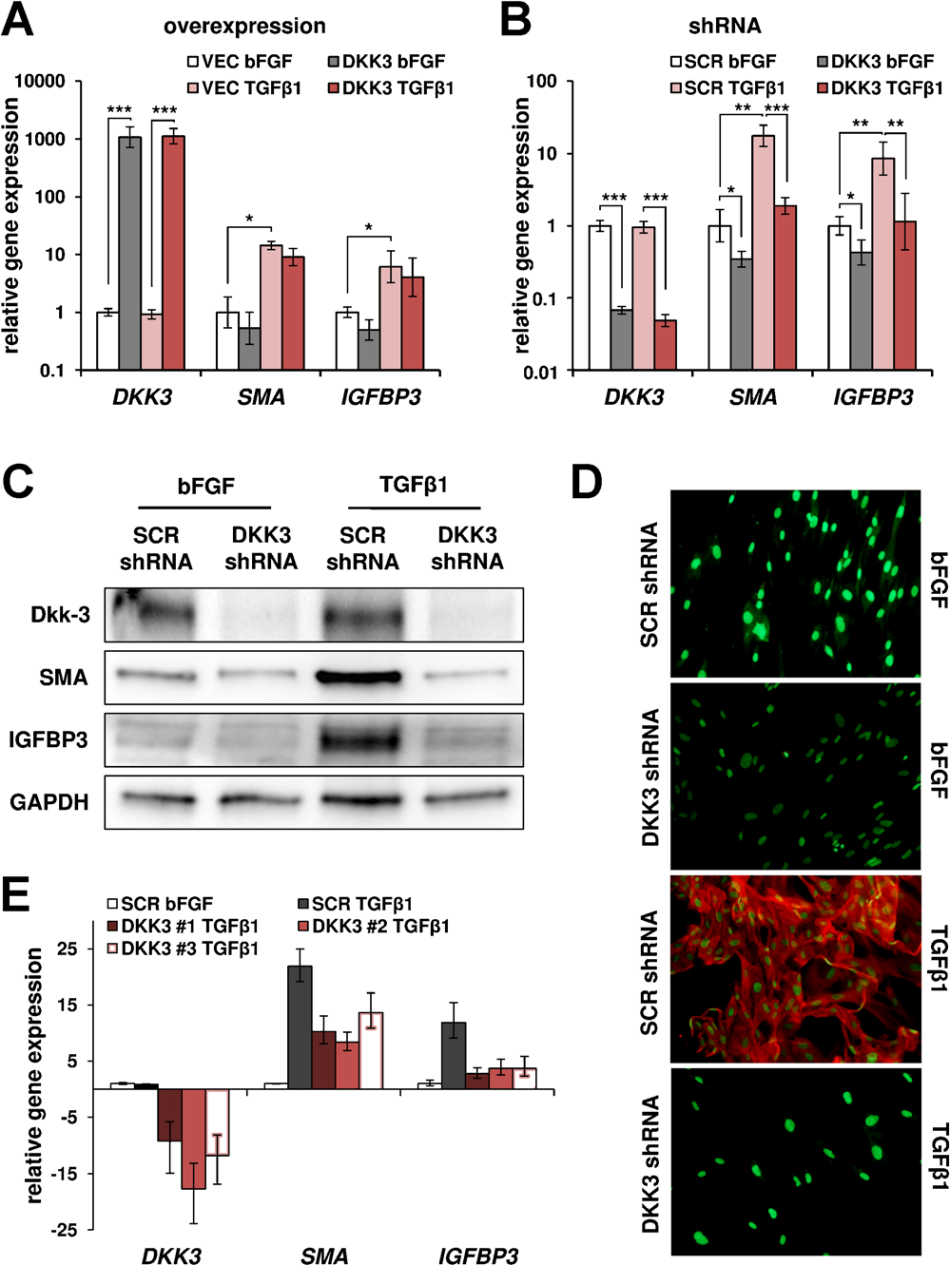
Knockdown of *DKK3* suppresses fibroblast-to-myofibroblast differentiation. Seventy-two hours post-viral transduction PrSCs were stimulated with 1 ng/ml TGFβ1 (myofibroblast differentiation) or bFGF (control) for 24 hrs. A: Lentiviral overexpression of *DKK3* (DKK3) did not affect TGFβ1-induced induction of mRNA levels of the myofibroblast differentiation markers *SMA* and *IGFBP3* compared with empty vector control (VEC). Bars represent mean ± SEM of four independent experiments. B: DKK3-shRNA significantly reduced basal mRNA levels of *SMA* and *IGFBP3* and suppressed TGFβ1-induced myofibroblast differentiation compared with scrambled control (SCR)-shRNA. Bars represent mean ± SEM of five independent experiments. C: Western blot analysis of SMA and IGFBP3 levels and D: immunofluorescence of SMA (red) in DKK3- and SCR-shRNA PrSCs after stimulation with bFGF or TGFβ1, respectively. E: Effect of siRNA-mediated *DKK3* knockdown using three different siRNA duplexes (DKK3 #1–#3) on *DKK3*, *SMA*, and *IGFPB3* mRNA levels compared with scrambled control siRNA (SCR) treated PrSCs (n = 3). C: GAPDH served as loading control. D: Nuclei were counterstained with SYTOX green.

The influence of Dkk-3 on TGFβ1-induced fibroblast-to-myofibroblast differentiation was assessed using the markers *SMA* and *IGFBP3* 27–28. In empty vector control PrSC treatment with TGFβ1 as expected led to significant induction of *SMA* (14.4 fold; *P* = 0.049) and *IGFBP3* (6.2-fold; *P* = 0.045) mRNA levels (Fig. 2A). Overexpression of *DKK3* neither significantly affected basal levels nor the potential of TGFβ1 to induce mRNA levels of both markers (Fig. 2A). Upon DKK3 knockdown however, basal mRNA levels of both markers were significantly attenuated in DKK3-shRNA PrSCs compared with SCR-shRNA control (Fig. 2B; *SMA*—2.9-fold; *P* = 0.034; *IGFBP3*—2.3-fold; *P* = 0.038). In SCR-shRNA cells TGFβ1 significantly induced *SMA* (17.6-fold; *P* = 0.003) and *IGFBP3* (8.6-fold; *P* = 0.005) levels, respectively, while differentiation was strongly suppressed in DKK3-shRNA PrSCs that expressed approximately basal mRNA levels of the control cells (Fig. 2B; *SMA* 1.9-fold; *P* vs. TGFβ1-treated SCR-shRNA = 0.00013; *IGFBP3* 1.1-fold; *P* = 0.0094). These findings were confirmed at the protein level by Western blot analysis for SMA and IGFBP3 (Fig. 2C) and immunofluorescence for SMA (Fig. 2D). Of note, TGFβ1-treatment did not significantly affect Dkk-3 mRNA or protein levels compared to bFGF control treated PrSCs (Fig. 2A–C).

To exclude potential off-target effects of the lentiviral-delivered DKK3-shRNA construct a set of three different *DKK3*-targeted siRNA duplexes was investigated. As observed with DKK3-shRNA, siRNA-mediated *DKK3* knockdown in PrSCs attenuated TGFβ1-induced fibroblast-to-myofibroblast differentiation as determined by *SMA* and *IGFBP3* mRNA levels compared with scrambled control siRNA-treated cells (Fig. 2E).

### Dkk-3 Attenuates Expression of Angiopoietin-1 in PrSCs

**Figure 3:**
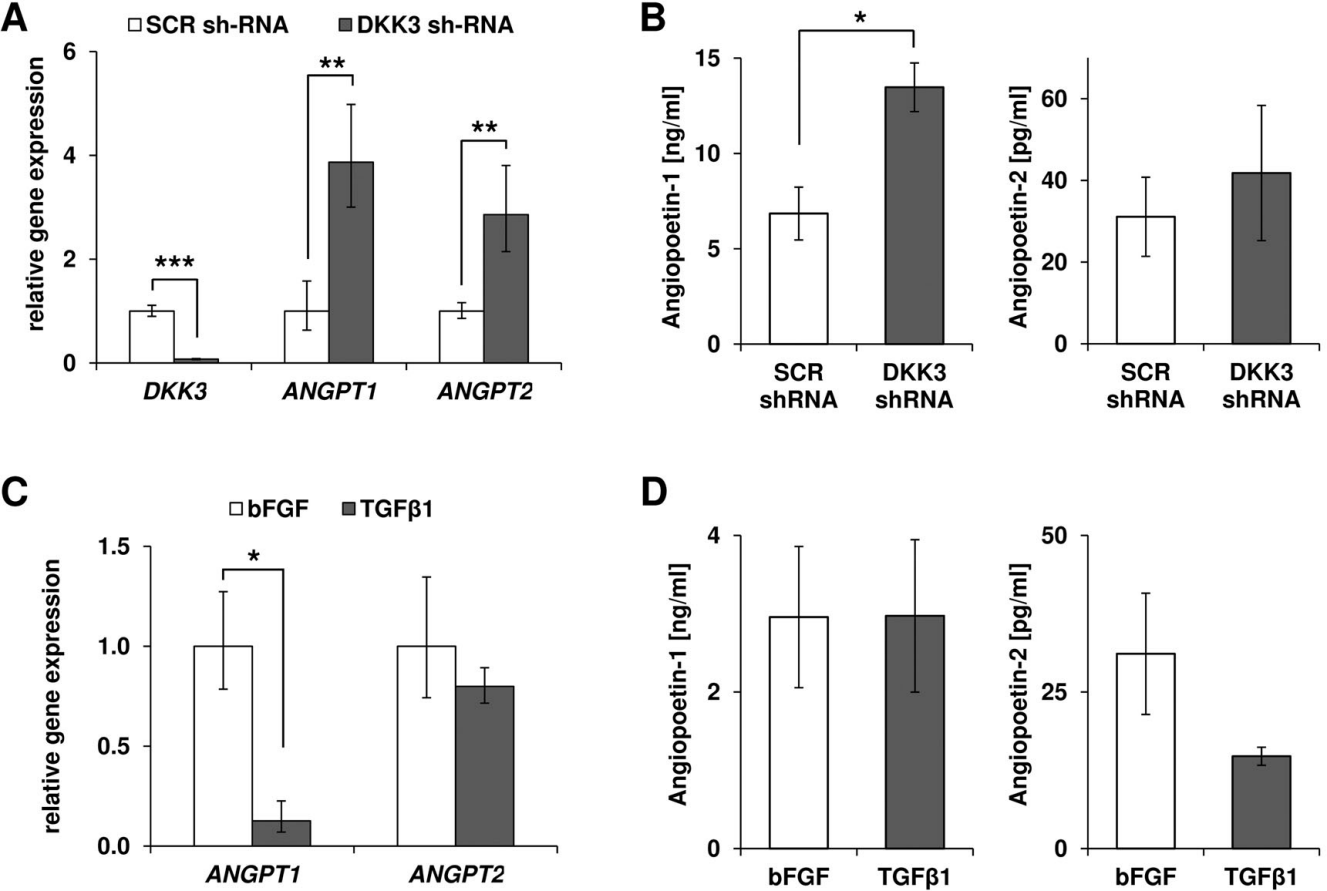
Knockdown of *DKK3* induces expression and secretion of ANGPT1. A: Lentiviral-delivered DKK3-shRNA in PrSCs led to a significant induction of *ANGPT1* and *ANGPT2* mRNA levels as determined by qPCR 72 hr post-transduction. Gene expression levels were normalized using the housekeeping gene *HMBS* and are shown relative to lentiviral-delivered scrambled control (SCR) shRNA. B: Secreted angiopoietin-1 levels were significantly elevated in DKK3-shRNA compared with SCR-shRNA PrSCs, while angiopoietin-2 protein levels were unaffected. C: mRNA and D: secreted protein levels of ANGPT1 and ANGPT2 in PrSCs determined after stimulation with 1 ng/ml TGFβ1 (myofibroblast differentiation) or bFGF (control) for 24 hr. Bars represent mean ± SEM of three independent experiments.

The impact of Dkk-3 on expression of angiopoietin-1 and angiopoietin-2 was analyzed by qPCR and ELISA. *ANGPT2* was approximately 500-fold less expressed compared to *ANGPT1* in PrSCs at mRNA levels (Supplemental Fig. 2). shRNA-mediated knockdown of *DKK3* resulted in elevated mRNA levels of both *ANGPT1* (3.9-fold; *P* = 0.0014) and *ANGPT2* (2.9-fold; *P* = 0.0063) compared with SCR-shRNA PrSCs expressing endogenous Dkk-3 levels (Fig. 3A), indicating that Dkk-3 represses the expression of angiogenic factors in PrSCs. Consistently, secreted angiopoietin-1 levels were significantly elevated in DKK3-shRNA PrSCs (Fig. 3B; 13.5 ± 1.3 vs. 6.8 ± 1.4 ng/ml; *P* = 0.048), while in contrast secreted angiopoietin-2 levels were not affected by *DKK3* knockdown (Fig. 3B; 41.8 ± 16.5 vs. 31.1 ± 9.7 pg/ml; *P* = 0.29). However, similar to mRNA levels, secreted angiopoietin-2 protein levels were very low compared to angiopoietin-1, indicating that PrSCs are not likely to significantly affect overall angiopoetin-2 levels in vivo.

The changes in expression levels of these angiogenic factors were investigated during myofibroblast differentiation (Fig. 3C and D). TGFβ1-induced fibroblast-to-myofibroblast differentiation significantly reduced mRNA levels of *ANGPT1* (−7.9fold; *P* = 0.013) but neither affected *ANGPT2* mRNA nor secreted angiopoetin-1 (2.97 ± 0.97 ng/ml vs. 2.96 ± 0.90 ng/ml; *P* = 0.98) and angiopoetin-2 (14.7 ± 1.4 ng/ml vs. 31.1 ± 9.7 pg/ml; *P* = 0.20) protein levels compared to bFGF-treated control cells.

### DKK3 Knockdown Attenuates PI3K/AKT Signaling But Does Not Affect Wnt/β-Catenin Signaling

**Figure 4:**
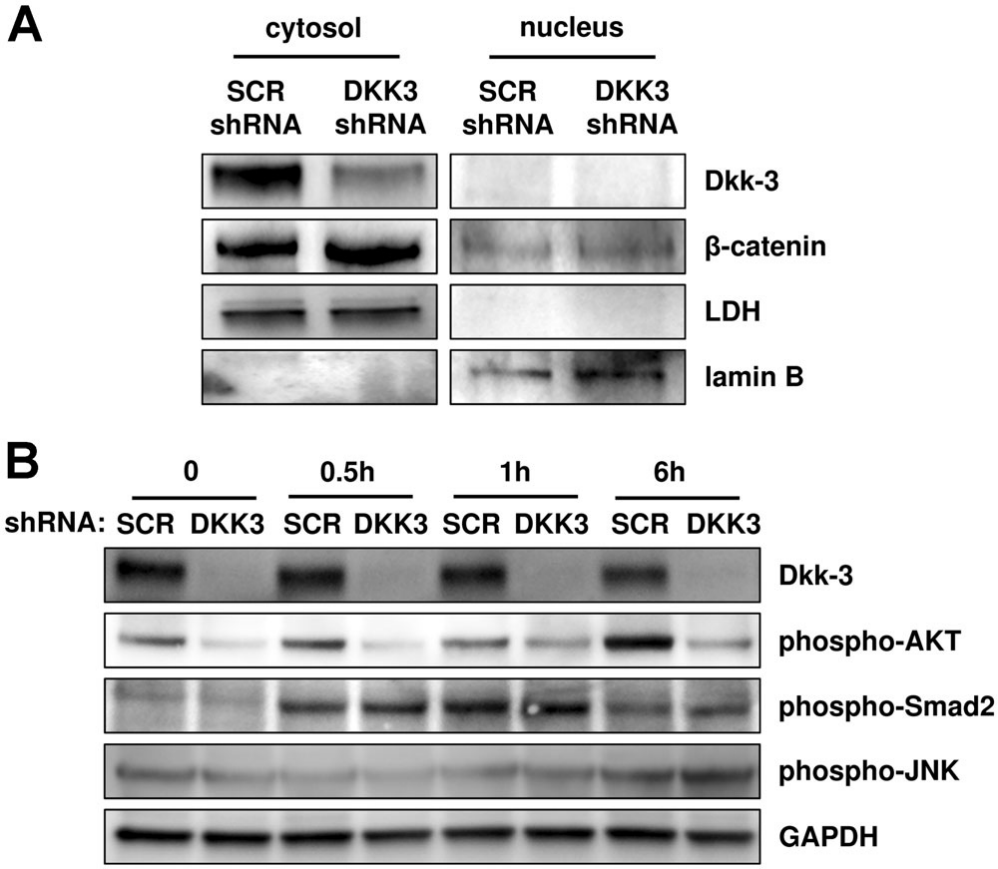
*DKK3* knockdown in PrSCs attenuates phosphorylation of AKT during differentiation. A: Lentiviral-delivered shRNA-mediated *DKK3* knockdown (DKK3 shRNA) affected neither cytosolic nor nuclear β-catenin levels compared with lentiviral-delivered scrambled control (SCR) shRNA, as determined by Western blot analysis 72 hr post-transduction. LDH and lamin B served as cytoplasmic and nuclear loading controls, respectively. As a secretory glycoprotein, Dkk-3 was localized in the cytosolic fraction and efficiently downregulated at protein levels by DKK3-shRNA. B: Western blotting of lysates from DKK3 and SCR shRNA-treated PrSCs stimulated with TGFβ1 for the indicated time with the antibodies shown. GAPDH served as loading control.

We next analyzed potential molecular pathways by which Dkk-3 mediates its effects. Since Dkk-3 has been related to Wnt/β-catenin signaling, the effect of DKK3-shRNA on β-catenin localization was analyzed using subcellular fractions. However, DKK3-shRNA neither affected cytosolic nor nuclear β-catenin levels in PrSCs (Fig. 4A), indicating that the effects of DKK3-shRNA are unlikely to be mediated via Wnt/β-catenin signaling.

Subsequently, we investigated the PI3K/AKT signaling pathway, a known mediator of proliferation and angiogenesis. DKK3-shRNA reduced basal AKT phosphorylation in PrSCs compared to SCR-shRNA treated control cells and additionally strongly attenuated induction of AKT phosphorylation in response to TGFβ1 treatment (Fig. 4B), raising the possibility that the aforementioned modulatory effects of DKK3-shRNA on differentiation and angiogenic markers were due to attenuation of PI3K/AKT signaling.

We additionally analyzed phosphorylation of the TGFβ-signaling mediator Smad2 and c-jun N-terminal kinase (JNK) which is essential for myofibroblast differentiation 29. However, DKK3-shRNA did not significantly affect phosphorylation of both, Smad2 or JNK (Fig. 4B).

### Inhibition of PI3K Mimics the Effects of DKK3 Knockdown

**Figure 5:**
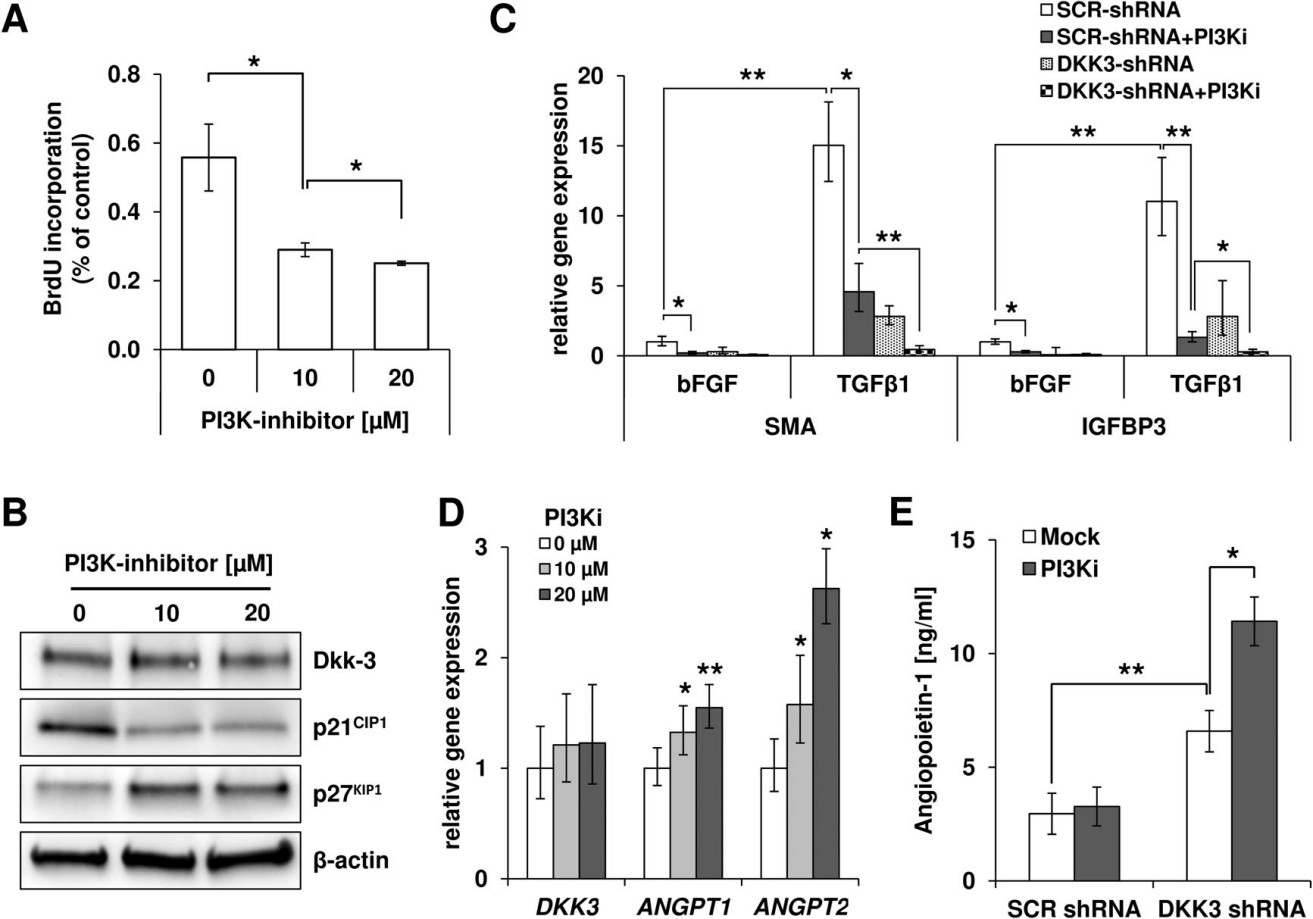
PI3K inhibition mimics the effects of *DKK3* knockdown. A: The PI3K inhibitor LY294002 reduced proliferation of PrSCs in a dose-dependent manner, as determined after 72 hr by BrdU-incorporation ELISA (n = 3). B: Western blot analysis of Dkk-3, p21^CIP1^ and p27^KIP1^ after 24 hr incubation with LY294002. β-actin served as loading control. C: Effect of PI3K inhibition using 10 µM LY294002 and/or lentiviral-delivered shRNA-mediated *DKK3* knockdown (DKK3-shRNA) on fibroblast-to-myofibroblast differentiation of PrSCs as determined by mRNA levels of the marker genes *SMA* and *IGFBP3* after stimulation with 1 ng/ml TGFβ1 (differentiation) or bFGF (control) for 24 hr. Gene expression levels were normalized using the housekeeping gene *HMBS* and are shown relative to scrambled (SCR-)shRNA and bFGF-treated controls. Bars represent mean ± SEM of three independent experiments. D: LY294002 induced *ANGPT1* and *ANGPT2* in a dose-dependent manner at mRNA levels within 4 hr of treatment (n = 3). E: Secreted angiopoietin-1 levels as determined in conditioned media of SCR-/DKK3-shRNA-treated PrSCs after incubation without/with 10 µM LY294002 for 72 hr (n = 4).

The specific PI3K inhibitor LY294002 was used to investigate whether attenuation of AKT phosphorylation in DKK3-shRNA PrSCs is responsible for the modulatory effects of *DKK3* knockdown on angiogenesis and fibroblast-to-myofibroblast differentiation marker expression. PI3K inhibition significantly attenuated cellular proliferation in a dose-dependent manner (Fig. 5A; 0 vs. 10 µM: *P* = 0.010; 10 vs. 20 µM: *P* = 0.044). Moreover, similar to DKK3-shRNA, the reduction in proliferation upon PI3K inhibition was associated with elevated *CDKN1B*/p27^KIP1^ mRNA and protein levels and reduced p21^CIP1^ protein levels while *CDKN1A* mRNA levels were increased (Fig. 5B and Supplemental Fig. 1). Dkk-3 levels were unaffected by LY294002 (Fig. 5B).

The influence of PI3K inhibition on TGFβ1-induced fibroblast-to-myofibroblast differentiation was assessed. As observed with DKK3-shRNA, 10 µM LY294002 significantly attenuated basal mRNA levels of the differentiation markers *SMA* (−4.9fold; *P* = 0.011) and *IGFBP3* (−3.5fold; *P* = 0.021) in SCR-shRNA PrSCs (Fig. 5C). Additionally, PI3K inhibition significantly suppressed TGFβ1-induced differentiation as determined at mRNA levels of *SMA* (4.6-fold vs. 15.0-fold; *P* = 0.027) and *IGFBP3* (1.3-fold vs. 11.0-fold; *P* = 0.0021), respectively, and combination of 10 µM LY294002 with DKK3-shRNA synergistically enhanced suppression of *SMA* (0.5-fold vs. 4.6-fold; *P* = 0.0039) and *IGFBP3* (0.3-fold vs. 1.3-fold; *P* = 0.033) induction by TGFβ1 (Fig. 5C).

Furthermore, gene expression of *ANGPT1* and *ANGPT2* was induced by inhibition of the PI3K/AKT signaling pathway in a dose-dependent manner. mRNA levels of *ANGPT1* (1.5-fold; *P* = 0.005) and *ANGPT2* (2.6-fold; *P* = 0.021) were significantly elevated after PI3K inhibition with 20 µM LY294002 (Fig. 5D). Treatment with 10 µM LY294002 slightly increased secreted angiopoietin-1 levels (Fig. 5E; 3.27 ± 0.85 ng/ml vs. 2.96. ± 0.90 ng/ml; *P* = 0.086) in SCR-shRNA PrSCs and synergistically enhanced elevation of angiopoietin-1 protein levels upon *DKK3* knockdown (Fig. 5E; 11.42 ± 1.07 ng/ml vs. 6.59. ± 0.91 ng/ml; *P* = 0.022). Angiopoietin-2 levels were not significantly affected by PI3K inhibition (Supplemental Fig. 2). Taken together, these data demonstrate that the effects of shRNA-mediated *DKK3* knockdown were mimicked and enhanced by PI3K/AKT inhibition.

## DISCUSSION

Based on elevated Dkk-3 expression in BPH and PCa-reactive stroma 1, the influence of Dkk-3 on remodeling of the tumor adjacent stroma was analyzed in vitro. Therefore lentiviral-delivered overexpression and shRNA-mediated knockdown of *DKK3* was applied to PrSCs.

In agreement with our previous finding using transient *DKK3* overexpression 1, stable overexpression of *DKK3* did not affect proliferation of PrSCs. These findings are in line with previous reports by ourselves and others that *DKK3* overexpression or addition of exogenous purified Dkk-3 protein failed to reduce proliferation or induce apoptosis in malignant cells 1–21. Moreover, these findings further support the hypothesis that reported anti-proliferative or pro-apoptotic effects of *DKK3* overexpression as a result of the unfolded protein response are in vitro artifacts that do not reflect the biological role of the endogenous protein.

We report herein that shRNA-mediated knockdown of *DKK3* significantly attenuated proliferation of PrSCs, a finding consistent with our previous observations in PANC-1 cells 21. While siRNA-mediated knockdown of *DKK3* in H460 lung cancer cells has been recently shown to cause apoptosis and increased levels of p53, p21^CIP1^ and BAX 39, we demonstrate that DKK3-shRNA mediated knockdown had no effect on phospho-p53 and BAX levels but increased p27^KIP1^ and reduced p21^CIP1^ levels. These effects could be mimicked by PI3K/AKT inhibition and are consistent with studies demonstrating stabilization of p21^CIP1^ by PI3K/AKT signaling at the protein level 40–41. Of note, both p27^KIP1^ and p21^CIP1^ mRNA levels were significantly elevated by DKK3-shRNA or PI3K/AKT inhibition (Supplemental Fig. 1), further suggesting elevated p21^CIP1^ protein degradation.

As determined by knockdown of endogenous *DKK3*, Dkk-3 supported fibroblast-to-myofibroblast differentiation, a central process of stromal remodeling that promotes the development of BPH and PCa. Dkk-3 has also been shown to support differentiation of other cell types. For example, depletion of Dkk-3 disrupted acinar morphogenesis of the prostate epithelial cell line RWPE-1 4–42. Moreover, Dkk-3 supported capillary formation of peripheral blood-derived endothelial colony-forming cells 34. On the other hand, DKK3-shRNA induced expression of differentiation markers in PANC-1 cells 21.

In vitro PrSCs abundantly expressed Dkk-3 whereas in the stromal compartment of the normal prostate Dkk-3 was not abundantly detected by immunohistochemistry 1. However, normal prostate tissue homeostasis is associated with slow turnover and low proliferation index of epithelial and stromal cells 43,44. Thus, it is conceivable that given the pro-proliferative activity of Dkk-3, in vitro out-growth of stromal cells from prostatic organoids selects for cells that (re-)express Dkk-3. Knockdown of *DKK3* in PrSCs might therefore reflect the quiescent homeostatic state of stromal cells associated with low proliferation and differentiation.

Interestingly, overexpression of *DKK3* did not affect TGFβ1-induced myofibroblast differentiation. Thus, Dkk-3 appears to be required as a permissive factor for efficient differentiation as well as proliferation, while its overexpression has no noticeable effect on PrSCs. This is consistent with a previously reported permissive role of Dkk-3 in TGFβ signaling during *Xenopus* mesoderm induction 46. In contrast, in RWPE-1 cells silencing of Dkk-3 increased TGFβ-signaling/phosphorylation of Smad-2 indicating that in epithelial cells Dkk-3 is not required as a permissive factor but rather limits TGFβ-signaling 42. However, in PrSCs phospho-Smad2 levels were unaffected by *DKK3* knockdown strongly suggesting that in stromal cells Dkk-3 does not directly regulate TGFβ/Smad-signaling. Dkk-3 expression was unaffected by TGFβ1-treatment, additionally excluding the possibility that Dkk-3 represents a downstream target of TGFβ1-signaling. In DKK3-shRNA PrSCs, suppression of TGFβ1-induced differentiation correlated with attenuated phosphorylation of AKT and inhibition of PI3K significantly attenuated myofibroblast differentiation, mimicking the effect of DKK3-shRNA. These findings suggest that Dkk-3 represents a permissive factor that supports proliferation as well as fibroblast-to-myofibroblast differentiation potentially via modulation of PI3K/AKT signaling. DKK3-shRNA did not significantly affect TGFβ-induced phosphorylation of Smad2 or c-jun N-terminal kinase, indicating that the effects of *DKK3* knockdown were specific to and mediated via AKT and not due to blocking upstream TGFβ1 signaling for example by attenuating global activation of the TGFβ receptor. However, the detailed mechanism how Dkk-3 enhances AKT phosphorylation remains unclear and future studies will focus on identifying Dkk-3-interacting partners and whether *DKK3* knockdown attenuates AKT phosphorylation upstream via PI3K or alternative kinases/phosphatases.

Given the conflicting data on a potential role of Dkk-3 in Wnt/β-catenin signaling 7–11 we investigated intracellular β-catenin levels that upon activation of the canonical Wnt signaling accumulates in the cytoplasm and is translocated into the nucleus. However, DKK3-shRNA did not affect β-catenin levels or subcellular localization, indicating that in PrSCs Dkk-3 does not act as a modulator of Wnt/β-catenin signaling.

In addition to inhibiting proliferation and differentiation, DKK3-shRNA or PI3K inhibition induced the expression of *ANGPT1* and *ANGPT2* mRNA and angiopoietin-1 but not angiopoetin-2 protein levels in PrSCs. *ANGPT2* has been shown to be induced by inhibition of PI3K/AKT signaling in endothelial cells 47. While *ANGPT1* is known to stimulate the PI3K/AKT pathway 48, our data indicate that *ANGPT1* expression is downregulated, potentially as a feedback loop, in response to PI3K/AKT. These findings raise the possibility that Dkk-3 is a co-factor in the initiation of the angiogenic switch observed in BPH and PCa that is associated with a shift in the ANGPT1/ANGPT2 ratio in favor of *ANGPT2*. We hypothesize that the loss of the vessel stabilizing factor *ANGPT1* (that is highly expressed in the absence of Dkk-3) due to elevated local Dkk-3 levels in endothelial cells and the surrounding stroma, leads to vessel destabilization that favors angiogenic sprouting.

Taken together our findings indicate that elevated Dkk-3 levels in the stromal compartment of BPH and PCa patients enhances (i) fibroblast proliferation and (ii) myofibroblast differentiation, both hallmarks of stromal remodeling and (iii) contribute to the angiogenic switch via suppression of vessel stabilizing angiogenic factors like *ANGPT1*.

## CONCLUSIONS

**Figure 6:**
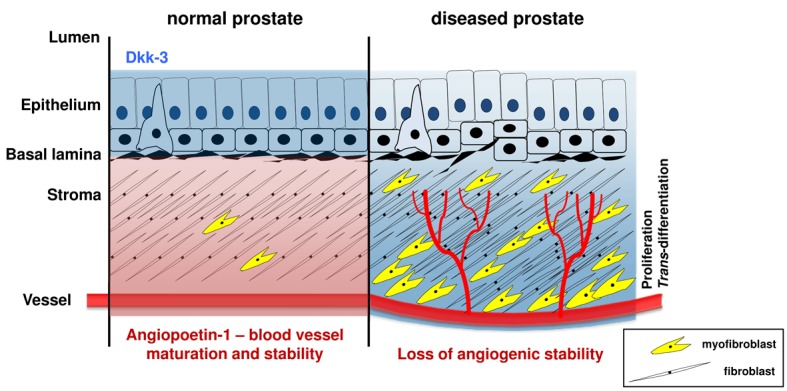
Proposed model of Dkk-3 impact on stromal remodeling in BPH and PCa. In normal prostate tissue, Dkk-3 is predominantly expressed in the epithelium but not in the stroma, which is characterized by a high fibroblast/myofibroblast ratio. Angiogenic factors such as ANGPT1, which stabilize the vessels, are highly expressed in endothelial and surrounding stromal cells that produce low levels of Dkk-3. In the diseased prostate, Dkk-3 expression is elevated in the stromal compartment, especially endothelial cells and potentially acts as a permissive factor for PI3K/AKT signaling, enhancing proliferation and differentiation of fibroblasts leading to stromal enlargement and elevated myofibroblast content. High Dkk-3 levels in vessels and surrounding stroma downregulate local expression of ANGPT1 shifting the ANGPT1/ANGPT2 ratio in favor of ANGPT2 that consequently results in vessel destabilization and sprouting of microvessels into the stroma.

Dkk-3 supported proliferation and fibroblast-to-myofibroblast differentiation and suppressed expression of angiogenic factors in PrSCs. DKK3-shRNA mediated knockdown attenuated AKT-phosphorylation and inhibition of PI3K mimicked the effects observed by *DKK3* knockdown, suggesting that Dkk-3 may represent a permissive co-factor of PI3K/AKT signaling in PrSCs. Collectively, these data suggest that altered Dkk-3 expression observed in BPH and PCa may support stromal proliferation and differentiation and the initiation of the angiogenic switch, all of which are key hallmarks of stromal remodeling in prostatic disease (Fig. 6). Therefore, Dkk-3 represents a potential therapeutic target for stromal remodeling in BPH and PCa.

## Abbreviations

ANGPT: angiopoietin
BAX: Bcl-2–associated X protein
BPH: benign prostatic hyperplasia
BrdU: bromodeoxyuridine
Dkk: Dickkopf-related protein
GAPDH: glyceraldehyde 3-phosphate dehydrogenase
HMBS: hydroxymethylbilane synthase
IEMA: immunoenymometric assay
IGFBP: insulin-like growth factor binding protein
JNK: c-jun N-terminal kinase
MOI: multiplicity of infection
PCa: prostate cancer
PI3K: phosphatidylinositol 3-kinase
PrEC: primary prostatic basal epithelial cells
PrSC: primary prostatic stromal fibroblasts
qPCR: quantitative PCR
SCR: scrambled
shRNA: short hairpin RNA
siRNA: small interfering RNA
SMA: smooth muscle cell actin

## Notes

Figure S1 A: *CDKN1A* (*P* = 0.0046) and *CDKN1B* (*P* = 0.0001) mRNA levels in primary prostatic stromal cells (PrSCs) were elevated upon *DKK3* knockdown using lentiviral-delivered shRNA compared to srambled control (SCR) shRNA as determined 72 hr post-transduction (n = 6). B: Treatment with 10 mM of the PI3K inhibitor LY294002 for 4 hr induces *CDKN1A* (*P* = 0.0036) and *CDKN1B* (*P* = 0.0067) mRNA levels in PrSCs (n = 5). Gene expression levels were normalized using the housekeeping gene *HMBS* and are shown relative to controls.

Figure S2 A: Basal *DKK3*, *ANGPT1*, and *ANGPT2* mRNA levels in primary prostatic stromal cells (PrSCs; n = 3) relative to the housekeeping gene *HMBS*. B: 72 hr post-viral transduction with DKK3-shRNA or scrambled control (SCR) shRNA PrSCs were stimulated with 1 ng/ml bFGF or TGF b _ 10 mM of the PI3K inhibitor LY294002 for 72 hr. Secreted angiopoietin-2 levels were not significantly affected by any treatment applied (n = 3).
