# Supplementary figures and images for: Dickkopf-related protein 3 promotes pathogenic stromal remodeling in benign prostatic hyperplasia and prostate cancer
Source: Prostate. 2013 Jun 14;73(13):1441–52. doi: 10.1002/pros.22691 (PMC3842835; doi:10.1002/pros.22691)

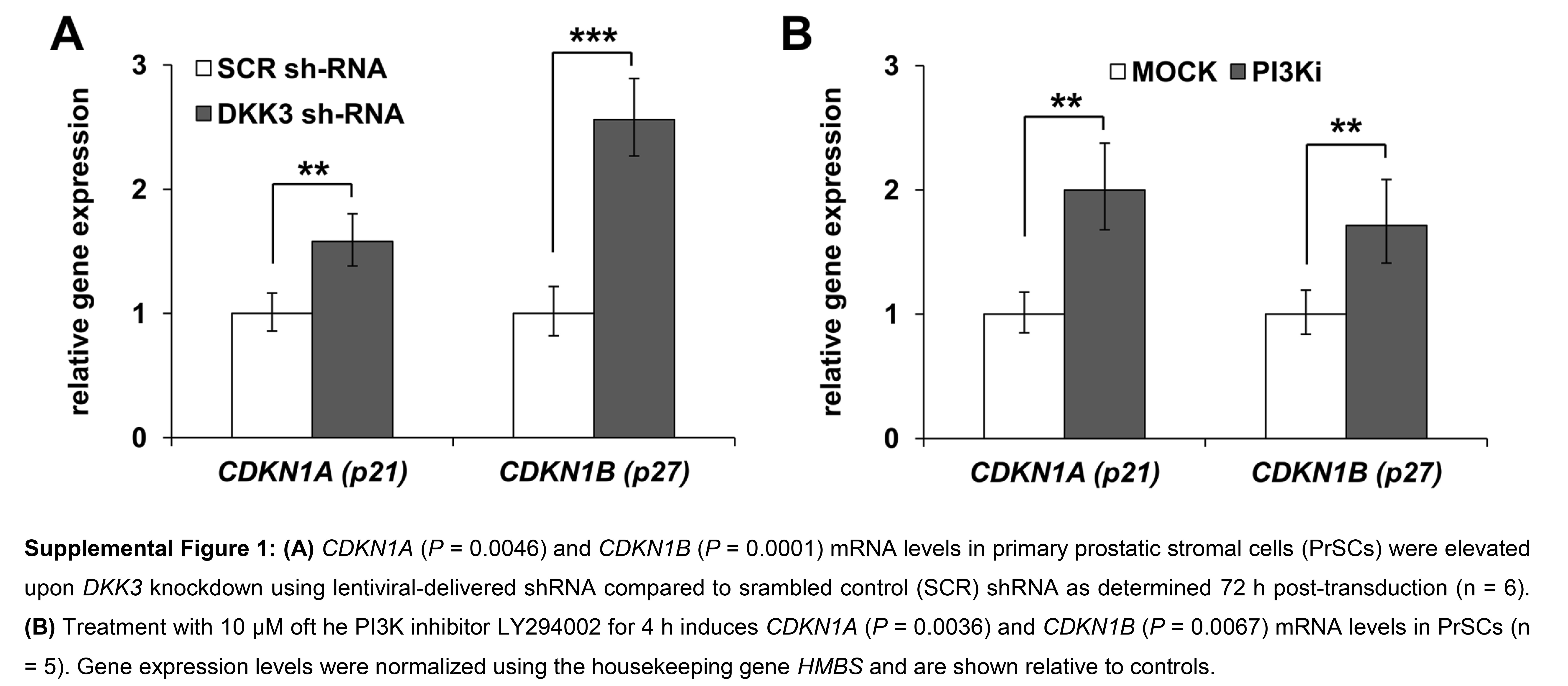

Supplement: Figure S1 — A: CDKN1A (P = 0.0046) and CDKN1B (P = 0.0001) mRNA levels in primary prostatic stromal cells (PrSCs) were elevated upon DKK3 knockdown using lentiviral-delivered shRNA compared to srambled control (SCR) shRNA as determined 72 hr post-transduction (n = 6). B: Treatment with 10 mM of the PI3K inhibitor LY294002 for 4 hr induces CDKN1A (P = 0.0036) and CDKN1B (P = 0.0067) mRNA levels in PrSCs (n = 5). Gene expression levels were normalized using the housekeeping gene HMBS and are shown relative to controls. [file pros0073-1441-sd1.tif]

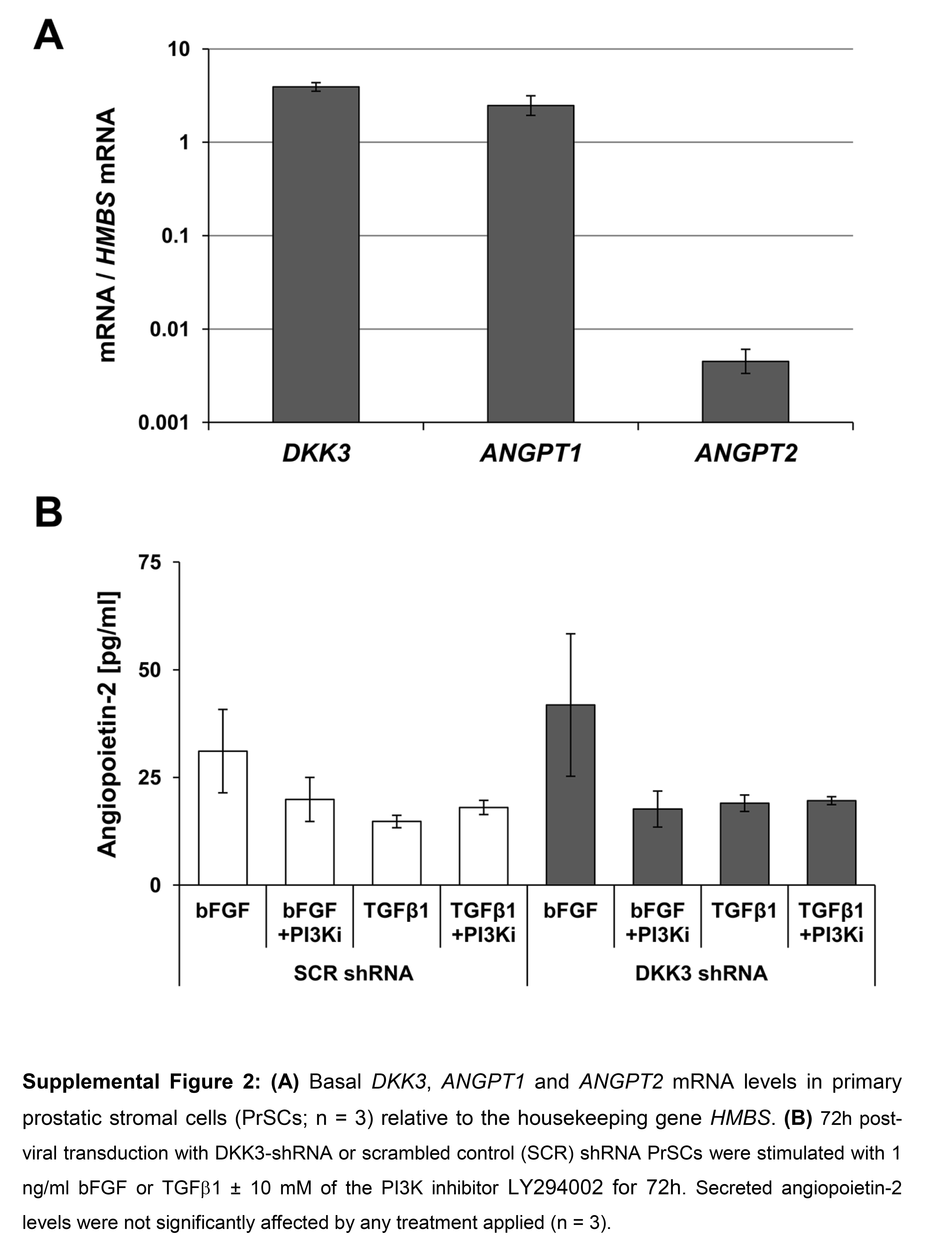

Supplement: Figure S2 — A: Basal DKK3, ANGPT1, and ANGPT2 mRNA levels in primary prostatic stromal cells (PrSCs; n = 3) relative to the housekeeping gene HMBS. B: 72 hr post-viral transduction with DKK3-shRNA or scrambled control (SCR) shRNA PrSCs were stimulated with 1 ng/ml bFGF or TGF b _ 10 mM of the PI3K inhibitor LY294002 for 72 hr. Secreted angiopoietin-2 levels were not significantly affected by any treatment applied (n = 3). [file pros0073-1441-sd2.tif]
